# Draft Genome Sequence of *Paracoccus* sp. Strain 228, Isolated from Surface Water of the Gulf of Gdańsk in the Baltic Sea

**DOI:** 10.1128/MRA.00347-19

**Published:** 2019-07-18

**Authors:** Joanna Karczewska-Golec, Maja Kochanowska-Łyżen, Magdalena Bałut, Arkadiusz Piotrowski, Piotr Golec, Agnieszka Szalewska-Pałasz

**Affiliations:** a Laboratory of Environmental Pollution Analysis, Faculty of Biology, University of Warsaw, Warsaw, Poland; b Department of Bacterial Molecular Genetics, University of Gdańsk, Gdańsk, Poland; c Department of Biology and Pharmaceutical Botany, Medical University of Gdańsk, Gdańsk, Poland; d Department of Applied Microbiology, Institute of Microbiology, Faculty of Biology, University of Warsaw, Warsaw, Poland; University of Maryland School of Medicine

## Abstract

We present here the draft genome sequence of Paracoccus sp. strain 228, isolated from the Gulf of Gdańsk in the southern part of the Baltic Sea. The assembly contains 4,131,609 bp in 32 scaffolds.

## ANNOUNCEMENT

The Paracoccus genus (*Rhodobacteraceae* family) contains versatile species adapted to various aquatic and terrestrial habitats. These Gram-negative metabolically flexible bacteria utilize different substrates, making them suitable for use in bioremediation (1, 2). Their tolerance for diverse growth conditions includes salt concentration (0 to 8% NaCl), temperature (7 to 42°C), and possible switch to anaerobic growth (3–7). The Baltic Sea is known for its altering conditions, and gulfs are especially susceptible to physicochemical parameter variations (8, 9). An understanding at the molecular level of the bacterial adaptation to this specific environment is important for marine research.

Paracoccus sp. strain 228 was isolated from the surface water of the Gulf of Gdańsk (in the Baltic Sea), as described previously (10). The whole-genomic DNA was isolated using the Sigma GenElute bacterial genomic DNA kit from bacteria cultured in Instant Ocean medium. For Illumina HiSeq 2000 platform sequencing, shotgun and 3-kb mate pair libraries were generated (using the Nextera mate pair library gel-plus kit) and sequenced with 2 × 101-bp reads, providing a total of 26,398,898 reads. After a quality check (FastQC version 0.10.1; http://www.bioinformatics.babraham.ac.uk/projects/fastqc/), error correction, and filtering (in which a minimal base quality of Q20 for 90% of the bases in each read of paired-end reads was applied), a total of 14,223,532 high-quality reads were retained, providing 146× average genome coverage. The genome processing and *de novo* assembly were performed using the SOAP*denovo*2 platform (11) at optimal *k*-mers (counted with Jellyfish version 1.1.10 [12]).

The genome assembly resulted in 32 scaffolds with an *N*_50_ scaffold size of 693,267 bp. The minimum and maximum scaffold lengths were 1,059 and 1,115,499 bp, respectively. The final assembly provided a draft genome containing 4,131,609 bp, with a high GC content (66.1 mol%) consistent with the average GC ratios of the *Paracoccus* genus (13). The draft genome annotation using the NCBI Prokaryotic Genome Annotation Pipeline (PGAP) version 2.9 (rev. 456657) (14) revealed 3,944 genes, with 3,491 predicted protein-coding sequences, 10 rRNA operons, and 51 tRNA genes. Moreover, two cryptic prophage sequences and one potentially functional prophage sequence were identified by PHASTER (15, 16).

The genome neighbor analysis of draft and complete genome sequences from NCBI databases revealed two closest sequences, those of *Paracoccus* sp. strain Arc7-R13 (82.08% symmetrical and 97.4% gapped identity) and *Paracoccu*s sp. strain S4493 (78.77% symmetrical and 97.33% gapped identity).

The newly assembled *Paracoccus* sp. 228 genome would provide input in studies on the adaptation of marine bacteria to their habitat. Analysis of its genomic potential revealed the presence of numerous stress response genes (e.g., those involved in compatible solute synthesis and osmotic and oxidative stress), membrane transport mediators, a functional motility system, and a higher-than-average abundance of genes involved in energy acquisition and macromolecule synthesis facilitating metabolic flexibility. Detailed molecular study of the adaptive processes in *Paracoccus* sp. 228 will be performed using transcriptomic and proteomic approaches.

### Data availability.

This whole-genome shotgun project has been deposited at DDBJ/EMBL/GenBank under the accession number JYGY00000000. The version described in this paper is the first version, JYGY01000000. The Sequence Read Archive (SRA) accession number is SRR8793599.
